# Bright Light Therapy in Psychiatric Disorders: Mechanisms, Clinical Procedures and Evidence

**DOI:** 10.3390/life16030449

**Published:** 2026-03-10

**Authors:** Simone Pardossi, Letizia Bossini, Veronica Milani, Maria Beatrice Rescalli, Alessandro Cuomo

**Affiliations:** Department of Molecular Medicine, University of Siena School of Medicine, 53100 Siena, Italy; letizia.bossini@gmail.com (L.B.); v.milani1@student.unisi.it (V.M.); m.rescalli@student.unisi.it (M.B.R.); alessandro.cuomo@unisi.it (A.C.)

**Keywords:** bright light therapy, mood disorders, circadian rhythms

## Abstract

Light is the primary zeitgeber for circadian rhythms, and also through these mechanisms, is closely related to mood regulation. Bright light therapy (BLT) is a therapeutic intervention that specifically exploits this physiological mechanism. This review summarizes the clinical procedures of BLT, the mechanisms through which light influences circadian rhythms and mood, and the evidence supporting BLT in psychiatric disorders. BLT is administered by considering device distance, treatment duration, and light intensity. Through pathways originating in the retina and projecting to the Suprachiasmatic Nucleus (SCN), light might generate signals within the central nervous system that influence not only circadian regulation but also mood, via connections involving the limbic system, the lateral habenula, and interactions with the hormonal system. At the clinical level, the strongest evidence for BLT concerns seasonal affective disorder, but data also indicate antidepressant efficacy in major depressive disorder and bipolar disorder, with an excellent tolerability profile. Emerging evidence further suggests benefits for insomnia, and sporadic and heterogeneous findings have explored its potential role in other conditions. Future studies are needed to better define the role of BLT in additional psychiatric disorders and in specific symptom domains that may not adequately respond to standard treatments, such as sexual dysfunction.

## 1. Introduction

Light is the fundamental *zeitgeber* for circadian timing in the central nervous system (CNS) timing, constituting the primary external signal for aligning the timing of endogenous biological processes [1,2]. Indeed, light acts as a biological metronome, conferring regularity and stability to circadian rhythms and preventing their desynchronization from the 24-h day [3]. This influence extends beyond sleep–wake regulation to multiple other physiological and behavioral functions; therefore, it is not surprising that light also plays a fundamental role in mood regulation [4].

Epidemiological studies show that mood is influenced by geographic latitude, with a higher prevalence of seasonal mood disorders, greater seasonal symptom variability, and poorer affective outcomes at increasing distances from the equator [5]. This latitudinal gradient is closely related to differences in light exposure and photoperiod variability, which increase markedly at higher latitudes and are minimal near the equator, where daylight remains relatively stable throughout the year [5]. Notably, in patients with bipolar disorder, greater photoperiod variability has been associated with a higher likelihood that the polarity of the first affective episode is depressive [6]. In bipolar disorder more broadly, variations in sunlight exposure have also been linked to other clinical features, including age at onset and suicide attempts [6,7]. Moreover, when considering environmental determinants of mood, it is also important to acknowledge the potential role of climate change, which has reshaped patterns of daylight exposure through changes in cloud cover, precipitation, and extreme weather events, potentially contributing to mental health risk in vulnerable individuals [8].

The clinical relevance of seasonality in psychiatric disorders is such that a specific specifier, “with seasonal pattern”, exists for both Major Depressive Disorder and Bipolar Disorder [9]. Given the clear association between mood and light exposure, bright light therapy (BLT)—which involves exposure to light of approximately 10,000 lux—has been extensively studied for affective disorders with a seasonal pattern [10]. The importance of this intervention is multifaceted. First, it directly targets a key hypothesized etiological factor underlying seasonal pattern disorders, namely, reduced light exposure [11]. Second, it represents an adjunctive approach to pharmacological treatment, given its feasibility as a standalone intervention [12] and as an augmentation strategy alongside medication [13]. In this context, antidepressants are often associated with adverse effects [14], among which sexual dysfunction is one of the most common [15]. BLT, in contrast, not only appears unlikely to cause such side effects, but preliminary studies suggest that it may even lead to improvements in sexual functioning [16].

In this non-systematic narrative review, we first describe the clinical and procedural aspects of light therapy, then outline the evidence on the mechanisms by which light regulates circadian rhythms and mood, and the data supporting the efficacy of BLT in psychiatric disorders.

Relevant literature was identified through targeted searches of PubMed and Google Scholar and by examining the reference lists of key articles. The search covered publications available up to December 2025 and was limited to articles published in English. For clinical evidence, we considered studies conducted in human populations, including randomized controlled trials, meta-analyses, observational studies, and other clinical investigations. For neurobiological mechanisms, experimental studies conducted in both human and animal models were also included. The selection of studies was guided by their relevance to the clinical and mechanistic scope of the review rather than by predefined inclusion criteria.

## 2. Procedural Aspects of Bright Light Therapy

To understand the technical features of BLT and how it is delivered, it is important to consider that treatment efficacy depends on light intensity, the distance between the device and the eyes, and the duration of exposure.

In general, the viewing distance should be set to achieve 10,000 lux at the corneal (eye) level, which depends on the specific device being used [10]. Across clinical studies, reported distances range from about 30 cm [17] up to 60 cm [18,19] or even 1 m [16]. The device is typically positioned at an oblique angle (e.g., about 30° from the line of gaze), and patients do not need to stare directly into the light source, but they must keep their eyes open so that light reaches the retina [10]. Not all trials specify the exact beam angle, although many describe placing the light box in front of the participant and slightly above eye level [20]. Still, experimental work in [21] indicates that the angle of light incidence can influence biological responses: one study compared two angles of exposure (28° vs. 55°) and found that the smaller angle produced a stronger effect on melatonin suppression, suggesting that incident angle may matter for circadian-relevant stimulation.

Regarding intensity at the corneal level, most clinical protocols use 10,000 lux [10,12] for 30 min, although lower intensities may also be effective when delivered for longer durations [10,22]. As noted above, exposure duration should be inversely proportional to intensity: protocols using 10,000 lux are often delivered for about 30 min, whereas sessions typically need to be extended to 1–2 h when using 2500 lux [10,22]. The key concept is that the therapeutic “dose” depends largely on the product of intensity and duration. For instance, in a trial in patients with bipolar depression using 7000 lux, the duration was slowly titrated upward with the goal of improving tolerability and reducing adverse effects [23].

With regard to spectral composition, short-wavelength (“blue-enriched”, ~480 nm) light has been proposed to produce stronger biological effects because melanopsin-containing intrinsically photosensitive retinal ganglion cells (ipRGCs) show peak sensitivity in the blue range of the spectrum [24]. However, clinical evidence does not consistently demonstrate the superior antidepressant efficacy of blue-enriched light compared with standard bright white light [25].

Finally, timing varies by condition and study design. In SAD trials, BLT is commonly administered early in the morning, shortly after awakening [16,26]. The optimal timing may also depend on chronotype: one trial scheduled light exposure based on the individual’s estimated dim light melatonin onset (DLMO) [27]. In contrast, the bipolar trial above-mentioned delivered BLT at midday (approximately 12:00–14:30) [23].

BLT is also administered in patients who are already receiving psychopharmacological treatment or psychotherapy [13,28,29,30].

It is not entirely clear why such light intensities and exposure durations are required, particularly considering that these levels exceed what is needed to elicit several non-image-forming responses to light under controlled laboratory conditions, such as melatonin suppression or circadian phase shifting, which can occur at substantially lower illuminances [31]. However, two aspects should be taken into consideration. First, photopic lux is a metric optimized for cone-mediated visual perception and does not adequately quantify melanopsin-weighted stimulation ipRGCs, which are central to many non-image-forming responses to light [24]. Second, in real-world conditions, several variables may influence the effective retinal light stimulus including ambient illumination, treatment adherence, viewing distance, and the position of the light source. This range of variability may therefore require higher nominal intensities and longer exposure durations.

## 3. Mechanisms of Action

**Figure 1:**
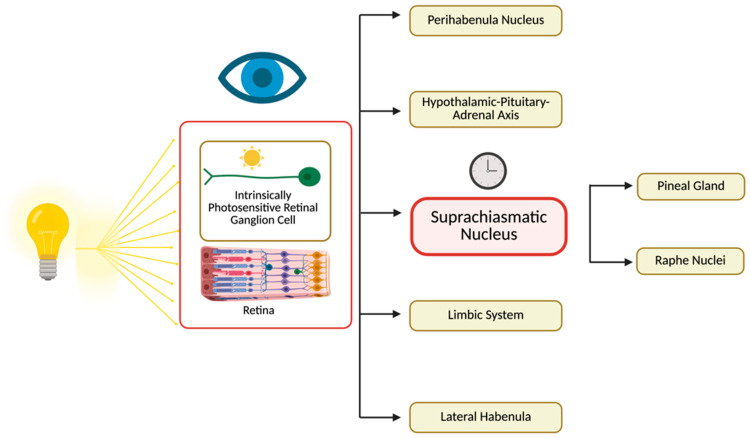
Retinal Light Input to Central Circadian and Mood-Regulating Pathways. Light signals captured by intrinsically photosensitive retinal ganglion cells are conveyed from the retina to the suprachiasmatic nucleus and interconnected brain systems, coordinating circadian timing and mood-related regulation.

Light can influence circadian rhythms and mood by acting on distinct anatomical structures, with signaling pathways originating in the retina (Figure 1). In the following paragraphs, we describe the specific mechanisms that link these anatomical targets to circadian and affective regulation. In the following paragraph, it should be taken into account that most mechanistic work relies on nocturnal rodent models, in which light may be aversive and where behavioral assays of “mood” may have limited translational specificity.

### 3.1. From the Retina to the Suprachiasmatic Nucleus Through Intrinsically Photosensitive Retinal Ganglion Cells (ipRGCs)

To clarify the potential effects of BLT on mood-related symptoms, it is necessary to understand the biological pathway of light from its initial receptor to its roles in circadian, neuroendocrine, and affective regulation. Firstly, the retina is not only a visual receiving organ but also the first element of a complex system that produces circadian and neuroendocrine responses [32,33]. Light entering the eye is detected by three major classes of photoreceptors: rods and cones, each with a specific function in producing visual images, as well as a discrete population of ipRGCs [32,33]. ipRGCs represent a specialized output of retinal neurons primarily dedicated to non-image-forming light responses [32]. They express melanopsin (OPN4), rendering them intrinsically photosensitive, with peak sensitivity to short-wavelength blue light (~480 nm), and they project directly to circadian and neuroendocrine brain centers, such as the Suprachiasmatic Nucleus (SCN) [32]. In addition to synaptic signals traveling from rods and cones to ipRGCs, ipRGCs themselves are able to respond directly to light intensity and spectral composition. This allows ipRGCs to mediate the biological effects of light in a course largely independent of conscious visual perception [32,34].

This hierarchical organization of the retina reflects a fundamental rule of retinal output. ipRGCs provide through their projections the major route—and under a variety of physiological conditions, possibly the dominant route—by which photic information enters non-image-forming brain systems. This has been demonstrated in several experimental paradigms showing that circadian, neuroendocrine, and behavioral responses to light can be largely preserved under some conditions, even in the absence of classical photoreception, whereas they are greatly reduced or lost entirely when melanopsin signaling by ipRGCs is interrupted [33,35,36,37]. In rod–cone degenerate animal models, circadian photoentrainment and light-induced suppression of pineal melatonin are largely preserved [35]. It is important to distinguish between the loss of melanopsin signaling (e.g., *Opn4* knockout models) and loss of melanopsin-expressing ipRGCs themselves [36]. In *Opn4* knockout mice, non-image-forming responses are typically attenuated, but circadian entrainment under standard light–dark conditions can remain largely preserved, highlighting that extrinsic rod/cone input to ipRGC circuits can contribute to photic signaling even when melanopsin is absent. Conversely, selective ablation of melanopsin-expressing retinal ganglion cells leads to severe impairment of these responses [38]. These findings support the notion that rods and cones alone cannot explain the non-image-forming biological effects of light and that, under most physiological conditions, their influence on circadian and neuroendocrine systems is exerted through ipRGCs, which therefore represent the primary route by which retinal output reaches central targets [33,35,36,37].

At the cellular level, this gating role of ipRGCs can be attributed to the characteristics of melanopsin-based phototransduction [39]. Unlike rod–cone systems, melanopsin signaling is slow and sustained and comparatively resistant to adaptation [34], allowing ipRGCs to register absolute light intensity over longer periods rather than short-term changes in luminance [39]. This property is particularly important for circadian and neuroendocrine control, because it depends on cumulative rather than brief changes in brightness during exposure to light. Under continuous bright illumination, as in BLT, melanopsin-driven responses dominate ipRGC activity even when rod and cone inputs are experimentally reduced [24].

Once ipRGCs are activated, the most direct circadian pathway is their projection through the retinohypothalamic tract (RHT) to the SCN, which functions as the master circadian pacemaker coordinating daily rhythms of sleep–wake timing, endocrine secretion, core body temperature, and behavioral arousal [40]. Although the SCN integrates both photic and non-photic inputs, the RHT represents the primary pathway mediating the circadian effects of light relevant to BLT [41]. However, direct evidence demonstrating that the antidepressant effects of BLT are specifically mediated by pathways originating from ipRGCs is currently lacking, particularly considering that the discovery of these cells occurred after many of the early clinical studies on BLT had already been conducted: indeed demonstrating that ipRGC-driven signaling is necessary or sufficient, for clinical antidepressant effects of BLT in humans would require mechanistic patient studies that are largely not yet available.

### 3.2. Molecular Mechanisms in the Suprachiasmatic Nucleus (SCN)

**Figure 2:**
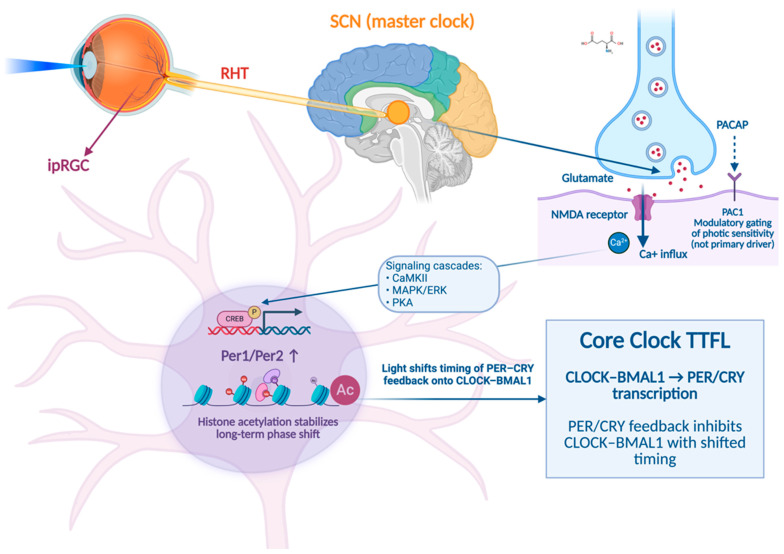
Mechanisms of action of light within the suprachiasmatic nucleus (SCN). Light information is detected by intrinsically photosensitive retinal ganglion cells (ipRGCs) in the retina and transmitted to the SCN via the retinohypothalamic tract (RHT), primarily through glutamate and pituitary adenylate cyclase-activating polypeptide (PACAP) signaling. This photic input modulates the molecular circadian clock by influencing the transcription–translation feedback loops involving circadian locomotor output cycles kaput (CLOCK), brain and muscle ARNT-like protein 1 (BMAL1), period (PER), and cryptochrome (CRY).

The mechanisms of light at the SCN level are illustrated in Figure 2. The RHT is not only a general sensory afferent but also a specialized circadian input pathway with a specific neurochemical code. At the synaptic level, retinal afferents to the SCN principally release glutamate as a fast excitatory transmitter, together with the neuropeptide pituitary adenylate cyclase-activating polypeptide (PACAP) [42,43].

Activation of N-methyl-D-aspartate (NMDA) receptors in SCN neurons rapidly leads to a rise in intracellular Ca^2+^, which in turn activates a series of calcium-sensitive signaling cascades, such as the calcium/calmodulin-dependent protein kinase II (CaMKII), mitogen-activated protein kinase/extracellular signal-regulated kinase (MAPK/ERK), and protein kinase A (PKA) pathways [44,45]. These cascades converge on nuclear transcriptional regulators, such as cAMP response element-binding protein (CREB), thereby regulating activity-dependent gene transcription [44,45]. Among the most robust transcriptional targets of this pathway are the core circadian clock genes *Per1* and *Per2*, whose light-induced expression represents a key molecular step leading to circadian phase resetting [44,45].

PACAP signaling, mediated primarily by PAC1 receptors, does not directly drive phase shifts, but modulates glutamate-induced responses by regulating intracellular cAMP levels and Ca^2+^ dynamics within SCN neurons [42,46]. Experimental studies have shown that disruption of PACAP signaling attenuates light-induced phase shifts and changes the shape of the phase response curve, indicating that PACAP plays a role in the temporal gating and sensitivity of photic entrainment rather than simply being an excitatory transmitter per se [43,46].

Beyond the initial glutamate–PACAP signaling, photic input to the SCN engages a broader network of intracellular and epigenetic mechanisms: calcium influx triggered by NMDA receptor activation leads not only to acute CREB phosphorylation, but also to sustained chromatin remodeling at clock gene promoters [42,47]. Light-induced histone acetylation at *Per1* and *Per2* loci has been demonstrated to facilitate transcriptional accessibility, thereby stabilizing phase shifts beyond the immediate photic stimulus [47,48].

In parallel, photic activation of the SCN modulates the balance between the positive and negative limbs of the core circadian transcription–translation feedback loop that underlies molecular timekeeping [45,49]. Under basal conditions, CLOCK–BMAL1 heterodimers drive the rhythmic transcription of *Period* (*Per*) and *Cryptochrome* (*Cry*) genes, while the delayed accumulation of PER–CRY protein complexes feeds back to inhibit CLOCK–BMAL1 activity, thereby generating a self-sustained ~24-h oscillation [50,51].

Light exposure transiently perturbs this equilibrium by inducing *Per* gene expression and increasing PER protein availability at specific circadian phases [45,49]. By altering the timing and dynamics of PER–CRY complex formation and nuclear translocation, photic input shifts the moment at which negative feedback is exerted on CLOCK–BMAL1-driven transcription. As a result, light does not simply trigger isolated changes in gene expression, but resets the phase of the entire molecular oscillator within the SCN [45,49]. This re-phasing of the core clockwork provides a mechanistic explanation for how brief or time-limited light exposure can translate into long-lasting changes in circadian timing and downstream physiological rhythms [45,49]. The relevance of understanding these molecular mechanisms within the SCN stems from the observation that the disruption of circadian rhythms is frequently reported in mood disorders and in psychiatric disorders more broadly [52]. In this context, genome-wide association studies of chronotype—a behavioral proxy for circadian timing—have identified loci associated with chronotype, including several core circadian clock genes (e.g., *PER* and *BMAL1*), supporting a genetic contribution of circadian biology to inter-individual differences in circadian timing [53]. Moreover, epidemiological studies have also shown associations between chronotype and depression risk [54]. The direction of the phase shift depends strongly on when light reaches the SCN. Human laboratory studies mapping the phase response curve (PRC)—that is, the systematic relationship between the circadian timing of light exposure and the resulting phase shift—demonstrate that bright light delivered during the early biological night induces phase delays, whereas light exposure during the late biological night or early morning produces phase advances [55]. This timing dependence supports the hypothesis that in individuals with delayed circadian phase or reduced rhythm robustness, appropriately timed BLT may contribute to symptom improvement via circadian realignment, which is particularly relevant for mood disorders characterized by delayed circadian phase or reduced rhythm robustness [56].

Furthermore, light-induced signaling cascades influence phosphorylation states of PER proteins through casein kinase pathways, thereby modifying protein stability, nuclear translocation, and degradation rates [50,51].

### 3.3. Downstream Circadian Effector Pathways: Melatonin and Monoaminergic Regulation

The molecular and transcriptional events described above within the SCN acquire systemic relevance through their downstream effector pathways, among which the regulation of pineal melatonin synthesis represents one of the most biologically and clinically relevant outputs.

Downstream of the SCN, one of the key circadian effector pathways for understanding the biological action of BLT is the control of pineal melatonin synthesis. At the molecular level, melatonin production in the pineal gland is regulated by sympathetic noradrenergic input, which activates β_1_-adrenergic receptors on pinealocytes, leading to increased intracellular cAMP levels and the induction of arylalkylamine N-acetyltransferase (AANAT), the rate-limiting enzyme in melatonin synthesis [57].

Photic activation of ipRGC–SCN signaling suppresses nocturnal melatonin production by inhibiting the multisynaptic sympathetic pathway linking the SCN to the pineal gland, which involves the paraventricular nucleus, the intermediolateral cell column of the spinal cord, and the superior cervical ganglion [57,58]. As a consequence, sympathetic noradrenergic input to the pineal gland is reduced, leading to rapid downregulation of AANAT activity and acute suppression of melatonin synthesis. Importantly, the circadian effects of BLT on melatonin are phase-specific rather than tonic. Morning light primarily advances melatonin offset, whereas evening or nighttime light delays melatonin onset. By reshaping the timing and duration of the melatonin signal, rather than simply reducing its amplitude, BLT contributes to the realignment of internal circadian phase relationships, particularly in individuals with delayed melatonin rhythms or reduced circadian amplitude [59,60].

In addition to regulating pineal melatonin synthesis, the SCN exerts circadian control over arousal and vigilance through projections to the serotonergic raphe nuclei (RN). Anatomical tracing and electrophysiological studies have shown that SCN output modulates the circadian pattern of serotonergic neuronal firing, thereby shaping daily variations in wakefulness and behavioral activation [61,62]. Conversely, serotonergic input to the SCN influences clock phase stability and photic responsiveness, as pharmacological or genetic manipulation of serotonin signaling alters light-induced phase shifts and circadian amplitude [63].

### 3.4. SCN-Independent Retinal Pathways Mediating Light Effects on Mood

Light also might exert effects on mood through retinal pathways that do not involve the SCN directly, but instead interact with limbic and monoaminergic circuits. Indeed, ipRGCs are a heterogeneous population that varies in projections and functions. M1-ipRGCs are endowed with high melanopsin expression and long-lasting light sensitivity, which gives them a particular preference for classical circadian targets such as the SCN and the perihabenular nucleus (pHb). M1-ipRGCs rely predominantly on intrinsic melanopsin-based phototransduction, whereas non-M1 subtypes depend more strongly on extrinsic synaptic input from rods and cones and therefore integrate both intrinsic and classical photoreceptor signals [39]. Non-M1 types, including M4-ipRGCs, however, show a broader range of projections to thalamic and limbic nodes involved in affective behavior regulation [33,64]. These subtype-specific retinal outputs produce distinct effects, as shown by high-level mapping studies and experimental manipulations, resulting in different behavioral responses to light. In mouse models, M4-ipRGCs project to the lateral habenula (LHb), a key control center for negative reward prediction and monoaminergic inhibition, via the ventral lateral geniculate nucleus (vLGN) and the intergeniculate leaflet (IGL) [65]. When this pathway is activated under bright light conditions during the daytime, it suppresses neuronal activity in the LHb itself, mainly through GABAergic inputs from the vLGN/IGL [65]. The LHb neurons targeted by this pathway project to midbrain monoaminergic centers including the dorsal raphe nucleus (serotonergic) and the ventral tegmental area (dopaminergic) [65]. By reducing stress-related LHb hyperactivity and burst firing, bright light may therefore disinhibit downstream serotonergic and dopaminergic signaling, providing a plausible circuit-level link between environmental light exposure and monoaminergic mechanisms implicated in depressive symptoms [65]. Importantly, selective stimulation or inactivation of this M4-ipRGC–vLGN/IGL–LHb circuit alone is sufficient to induce these effects, as shown by genetic and optogenetic manipulations [65]. In contrast, exposure to light during the biological night or under aberrant lighting conditions preferentially engages an alternative pathway originating from M1-ipRGCs [66]. The pHb lies at a hub between retinal inputs and further processing by forebrain regions such as the prefrontal cortex and the nucleus accumbens [66,67]. Activation of this network causes animals to exhibit depressive-like behavior, anhedonia, and abnormal reward processing, even in the absence of overt circadian disruption [66,67]. Thus, maladaptive light exposure may disrupt mood-regulatory systems through mechanisms that do not require overt changes in circadian phase or SCN lesions per se [66,67,68]. Together, these results show that the affective value of light is not only determined by timing but also by which type of ipRGC is engaged and how signals are routed within the habenular network.

ipRGCs also project to limbic structures, such as the bed nucleus of the stria terminalis and the medial amygdala, which are key nodes for the processing of anxiety and threat detection and response [37,69]. This organization of pathways provides a substrate for relatively rapid changes in emotional state induced by light, which cannot be easily inferred from circadian phase shifting or melatonin suppression alone and may contribute to the anxiolytic or activating effects of daytime light exposure [67].

Light can also influence mood through rapidly acting neuroendocrine pathways involving the hypothalamic–pituitary–adrenal (HPA) axis and the peripheral autonomic nervous system [70]. Human laboratory studies have shown that high-intensity light exposure can acutely alter cortisol secretion profiles, with effects varying by time of day, light intensity, and prior light history [70]. Experimental animal models have further shown that light can affect adrenal clock gene expression and glucocorticoid release through retinal pathways projecting to hypothalamic and brainstem autonomic centers [71,72]. While these studies identify plausible retina–limbic pathways by which light can modulate affect-related circuits, they do not by themselves establish that the same pathways mediate the clinical antidepressant effects of BLT in humans.

Although no studies have directly examined the clinical effects of BLT in humans through the retino-limbic pathways described above, the brain structures involved in these circuits have been widely studied in several psychiatric disorders. In particular, the lateral habenula has been associated with depressive states and altered reward processing [73], while dysregulation of the HPA axis is a well-established feature of stress-related disorders such as major depression and PTSD [74]. In addition, alterations in circadian clock genes, including *PER*, *CLOCK*, and *BMAL1*, as above-mentioned, have been linked to vulnerability to mood disorders [53].

## 4. Clinical Efficacy of BLT

Over the past three decades, BLT has been increasingly investigated as a non-pharmacological intervention for a range of psychiatric conditions, with the strongest and most consistent clinical evidence emerging in mood disorders (Table 1).

### 4.1. Depressive Disorders

The clearest and most dependable rapid clinical efficacy of BLT has been demonstrated in depressive states, particularly seasonal affective disorder (SAD). BLT has indeed been investigated for more than three decades, with early clinical reports dating back to the 1980s and comprehensive reviews published in the late 1990st [105]. Subsequent randomized trials and meta-analyses have generally supported its efficacy for seasonal affective disorder and, to a lesser extent, non-seasonal major depression. However, findings remain heterogeneous across studies, likely reflecting differences in treatment protocols (e.g., timing, light intensity, and duration), patient characteristics, and study design, highlighting the need for more standardized approaches in future trials. Since the earliest controlled trials, morning BLT has been shown to significantly reduce measures of depressive severity and to yield higher response rates compared with placebo or dim-light therapies [12,75,76]. Meta-analytic estimates generally indicate small-to-moderate effect sizes, yet with clinically meaningful rates of response and remission, comparable to those observed with first-line antidepressant medications in SAD [77].

Importantly, BLT efficacy is not limited to seasonal presentations [12,30]. In non-seasonal major depressive disorder, morning BLT has been shown to reduce depressive symptoms both as a stand-alone modality and as an augmentation strategy to pharmacological antidepressant therapy [30]. The superiority of morning BLT over placebo light in relieving depressive symptoms has been demonstrated [12], while subsequent studies confirmed additional benefits when BLT was administered concurrently with selective serotonin reuptake inhibitors or other antidepressant medications [13]. Meta-analytic syntheses consistently indicate higher odds of clinical response and remission with BLT compared with control conditions [78]. Beyond its combination with pharmacotherapy, BLT has also been studied in association with cognitive-behavioral psychotherapy, demonstrating efficacy in reducing depressive symptoms [106,107].

Evidence has also extended to specific clinical populations. In perinatal depression, BLT might reduce depressive symptom severity while avoiding pharmacological exposure to the fetus or infant, making it a potentially attractive non-pharmacological option. However, sample sizes remain small, and results are inconsistent, with substantial heterogeneity in control conditions, limiting the strength of conclusions that can be drawn at present [79,80]. In adolescent depression, BLT appears feasible and generally well-tolerated, with preliminary evidence indicating improvements in depressive symptoms as well as in both external and internal biological sleep–wake regulation [81].

### 4.2. Bipolar Disorders

Meta-analytic evidence has also highlighted the efficacy of BLT in bipolar disorder (BD), showing a reduction in depressive symptoms both in patients receiving pharmacological treatment and in those who are not, with a small-to-moderate but statistically significant antidepressant effect of BLT in bipolar depression [82,83,84]. Moreover, adjunctive BLT administered under mood-stabilizing treatment has not been associated with an increased risk of manic or hypomanic switch [82,83,84]. Most samples of the above-mentioned studies are predominantly composed of patients with bipolar I disorder, while bipolar II disorder is less represented. Nevertheless, subgroup analyses and descriptive data, together with more recent naturalistic studies [85], do not indicate differential efficacy or safety signals between bipolar I and bipolar II patients [82,83].

Treatment parameters appear to be critical, with light intensities ≥ 5000 lux and morning or midday administration being associated with the most consistent antidepressant effects [82]. A recent phase I/II randomized dose-escalation trial in bipolar I and II depression found that BLT delivered for up to 45 min per day was well-tolerated, elicited significant reductions in depressive symptoms, and was associated with a low incidence of hypomanic switching that resolved with dose adjustment [86]. This trial also observed a cumulative dose effect on global clinical improvement and suggested a potentially greater benefit with morning administration [86]. Circadian dysregulation is a well-established feature of bipolar disorder, with alterations in sleep–wake rhythms, chronotype, and melatonin regulation reported across different mood states [108]. Considering that BLT likely acts, at least in part, by restoring circadian rhythms [109], it could be hypothesized that this mechanism may contribute to its potential efficacy in bipolar disorder. However, in the absence of robust predictive biomarkers of treatment response, this remains largely speculative at present. Furthermore, recent real-world evidence, including both bipolar I and bipolar II patients, demonstrated that add-on BLT administered for one to two weeks was associated with significant reductions in self-reported depressive symptoms, with effect sizes comparable between bipolar and unipolar depression [85]. Improvements were pronounced in core depressive symptoms, such as mood, energy, and concentration, rather than in sleep, suggesting a possible mechanism of BLT that extends beyond circadian normalization. There were no detectable differences between bipolar subtypes [85].

### 4.3. Post-Traumatic Stress Disorder

BLT has also been studied in post-traumatic stress disorder (PTSD). A recent systematic review highlights that even though there were no observed significant differences between the treatment and the placebo groups regarding effects on sleep parameters, BLT was associated with improvements in PTSD symptom severity, as measured with the Clinician-Administered PTSD Scale for DSM-5 (CAPS-5) and fear extinction [87]. Across studies, BLT protocols typically involved morning exposure for 30–60 min over four to six weeks, with light intensities ranging from blue-enriched wavelengths at lower lux levels to standard bright white light at 10,000 lux [87].

BLT has also been shown to reduce reactivity in limbic regions implicated in anxiety and threat processing, including the insula and amygdala [88]. Structural neuroimaging data also indicate that BLT can increase left amygdala gray matter volume, with changes correlating with improvements in sleep quality and nightmare severity [89].

### 4.4. Other Psychiatric Conditions

BLT has also been investigated, albeit in a heterogeneous manner, across several other psychiatric disorders.

With regard to anxiety, considered either as an autonomous symptom domain or as a comorbid condition, evidence from clinical trials remains scattered and not yet well-structured. Although some studies have reported beneficial effects of BLT on anxiety symptoms, definitive conclusions cannot be drawn at present [90,91].

In adult ADHD, preliminary studies suggest possible improvements in core attentional symptoms, mood, and overall functioning following morning exposure to bright light, although the available data mainly derive from open-label designs or small samples [92]. Within the same diagnostic framework, one study reported improvements in ADHD symptoms by specifically targeting delays in sleep–wake rhythms through BLT, supporting a circadian-based therapeutic approach [93].

In eating disorders, the most extensively investigated condition is bulimia nervosa. Several studies have shown that morning BLT is associated with reduced binge-eating frequency and greater overall clinical improvement compared with control conditions, with more pronounced effects in patients with a seasonal pattern of symptoms [94,95]. Conversely, another trial demonstrated positive effects on mood but failed to show a significant reduction in binge-eating episodes [96]. Overall, evidence summarized in a systematic review suggests short-term benefits of BLT on eating-related and affective symptoms, albeit in the context of marked methodological heterogeneity and limited replicability across studies [97].

Finally, in schizophrenia spectrum disorders, small pilot studies have explored BLT as an adjunctive treatment, reporting some beneficial effects, particularly on negative symptoms [98,99], whereas another study did not observe significant improvements in these domains [100].

In the neurocognitive domain, several studies have investigated the efficacy of BLT for the management of agitation in dementia, with some reporting clinical benefits [101,102]. A crossover randomized controlled trial conducted in residential settings tested BLT to reduce agitated behaviors in patients with dementia and found signals of improvement specifically in agitated patients presenting with disrupted sleep–wake rhythms, but not in those without circadian alterations [103]. Consistently, meta-analytic evidence indicates that BLT exerts a greater impact on sleep-related disturbances in dementia populations than on agitation per se [104].

## 5. Conclusions and Future Perspectives

BLT is now a treatment supported by robust evidence for SAD. It represents an approach distinct from that of antidepressant medications, acting through regulatory pathways that directly involve circadian rhythms, which in turn influence mood. This approach may also have applications in other psychiatric disorders, at least as an augmentation strategy. However, several limitations remain: clinical studies have used heterogeneous treatment protocols with respect to timing, intensity, and duration of light exposure, which complicates direct comparison across trials. Moreover, the neurobiological mechanisms translating light exposure into clinical improvement are not yet fully understood. Beyond its efficacy on core affective symptoms (such as mood levels), BLT has also demonstrated beneficial effects on other dimensions of psychiatric presentations, including insomnia. Future studies could further investigate its role in addressing other common symptoms, such as sexual dysfunction, which not only often fail to respond to antidepressant treatments but may also emerge as adverse effects of these therapies.

Despite robust clinical efficacy in SAD and growing evidence in non-seasonal depression and bipolar depression, the biological mechanisms translating light exposure into symptom change remain incompletely defined. Future trials should incorporate standardized light metrics beyond photopic lux (including melanopic measures when feasible) and prospectively test whether changes in circadian markers (e.g., DLMO timing, amplitude/regularity measures) mediate or predict clinical response.

Overall, BLT may contribute to a more holistic approach to the treatment of psychiatric disorders, focusing not only on mood and anxiety but also on other symptomatic and functional domains, such as circadian rhythms and sleep, thereby adopting a more global perspective on psychopathology.

## Figures and Tables

**Table 1:** Summary of the available clinical evidence for bright light therapy across psychiatric disorders, including study type, typical populations, magnitude of effects, and strength of recommendation.

Indication	Type of Evidence	Typical Sample Characteristics	Nature of Effect Sizes
Seasonal Affective Disorder (SAD)	Multiple randomized controlled trials and meta-analyses [12,75,76,77]	Adults with recurrent winter depressive episodes	Small-to-moderate effect sizes with clinically meaningful response and remission rates
Major Depressive Disorder (non-seasonal)	Randomized controlled trials and meta-analyses [12,13,30,78]	Adults with non-seasonal MDD treated with BLT as monotherapy or augmentation	Small-to-moderate reductions in depressive symptoms
Perinatal Depression	Small randomized controlled trials [79,80]	Pregnant or postpartum women with depressive symptoms	Mixed findings with possible symptom reduction
Adolescent Depression	Pilot clinical studies [81]	Adolescents with major depressive disorder	Preliminary improvements in depressive symptoms and circadian regulation
Bipolar Depression	Meta-analyses, randomized trials, and naturalistic studies [82,83,84,85,86]	Bipolar I and II patients with depressive episodes	Small-to-moderate antidepressant effects with low manic switch risk under controlled protocols
Post-traumatic Stress Disorder (PTSD)	Systematic review and interventional studies (including neuroimaging trials) [87,88,89]	Adults with PTSD treated with morning BLT protocols	Improvements in PTSD symptom severity and fear extinction; limited or inconsistent effects on sleep parameters
Anxiety Symptoms	Small clinical trials and open-label studies [90,91]	Adults with primary or comorbid anxiety symptoms	Possible anxiolytic effects but heterogeneous evidence
Attention-Deficit/Hyperactivity Disorder (ADHD)	Open-label trials and pilot studies [92,93]	Adults with ADHD, often with delayed circadian phase	Improvements in attention, mood, and circadian alignment
Eating Disorders (Bulimia Nervosa)	Randomized controlled trials and systematic review [94,95,96,97]	Patients with bulimia nervosa	Mixed results on binge-eating frequency and mood symptoms
Schizophrenia Spectrum Disorders	Pilot clinical studies [98,99,100]	Patients with schizophrenia receiving BLT as adjunctive treatment	Possible improvement in negative symptoms; inconsistent findings
Dementia-related agitation and sleep disturbances	Randomized controlled trials and meta-analyses [101,102,103,104]	Older adults with dementia in residential care settings	Greater improvement in sleep and circadian disturbances than agitation

## Data Availability

No new data were created or analyzed in this study. Data sharing is not applicable to this article.

## Abbreviations

The following abbreviations are used in this manuscript:

BLT	Bright Light Therapy
SCN	Suprachiasmatic Nucleus
CNS	Central Nervous System
SAD	Seasonal Affective Disorder
MDD	Major Depressive Disorder
BD	Bipolar Disorder
PTSD	Post-Traumatic Stress Disorder
ADHD	Attention-Deficit/Hyperactivity Disorder
ipRGCs	Intrinsically Photosensitive Retinal Ganglion Cells
*OPN4*	Opsin 4 (melanopsin)
RHT	Retinohypothalamic Tract
DLMO	Dim Light Melatonin Onset
PRC	Phase Response Curve
NMDA	N-Methyl-D-Aspartate
CaMKII	Calcium/Calmodulin-Dependent Protein Kinase II
MAPK	Mitogen-Activated Protein Kinase
ERK	Extracellular Signal-Regulated Kinase
PKA	Protein Kinase A
CREB	cAMP Response Element-Binding Protein
PACAP	Pituitary Adenylate Cyclase-Activating Polypeptide
*PER*	Period (clock genes)
*CRY*	Cryptochrome (clock genes)
CLOCK	Circadian Locomotor Output Cycles Kaput
BMAL1	Brain and Muscle ARNT-Like 1
AANAT	Arylalkylamine N-Acetyltransferase
RN	Raphe Nuclei
LHb	Lateral Habenula
pHb	Perihabenular Nucleus
vLGN	Ventral Lateral Geniculate Nucleus
IGL	Intergeniculate Leaflet
HPA	Hypothalamic–Pituitary–Adrenal Axis
DSM-5	Diagnostic and Statistical Manual of Mental Disorders, Fifth Edition
CAPS-5	Clinician-Administered PTSD Scale for DSM-5
